# Comparison of Administration of 8-Milligram and 4-Milligram Intranasal Naloxone by Law Enforcement During Response to Suspected Opioid Overdose — New York, March 2022–August 2023

**DOI:** 10.15585/mmwr.mm7305a4

**Published:** 2024-02-08

**Authors:** Emily R. Payne, Sharon Stancliff, Kirsten Rowe, Jason A. Christie, Michael W. Dailey

**Affiliations:** ^1^AIDS Institute, New York State Department of Health; ^2^New York State Police, Albany, New York; ^3^Albany Medical College, Albany, New York.

**Summary**

**What is already known about this topic?**

In 2021, the Food and Drug Administration approved an 8-mg intranasal naloxone product, with twice the amount in the usual 4-mg dose; no data on use of this product in probable opioid overdoses are available.

**What is added by this report?**

Among recipients of 4-mg or 8-mg intranasal naloxone administered by law enforcement, no differences were observed in survival, the number of doses received, prevalence of most postnaloxone signs and symptoms, combativeness, or hospital transport refusal; 8-mg product recipients had a significantly higher prevalence of opioid withdrawal signs and symptoms than did 4-mg product recipients.

**What are the implications for public health practice?**

No benefits to administration of 8-mg intranasal naloxone compared with 4-mg product were found. More data are needed to determine whether higher-dose intranasal naloxone would provide added benefits.

## Abstract

In 2021, an 8-mg intranasal naloxone product was approved by the Food and Drug Administration; however, no studies have examined outcomes among persons who receive the 8-mg naloxone product and those who receive the usual 4-mg product. During March 2022–August 2023, New York State Department of Health (NYSDOH) supplied some New York State Police (NYSP) troops with 8-mg intranasal naloxone; other troops continued to receive 4-mg intranasal naloxone to treat suspected opioid overdose. NYSP submitted detailed reports to NYSDOH when naloxone was administered. No significant differences were observed in survival, mean number of naloxone doses administered, prevalence of most postnaloxone signs and symptoms, postnaloxone anger or combativeness, or hospital transport refusal among 4-mg and 8-mg intranasal naloxone recipients; however, persons who received the 8-mg intranasal naloxone product had 2.51 times the risk for opioid withdrawal signs and symptoms, including vomiting, than did those who received the 4-mg intranasal naloxone product (95% CI = 1.51–4.18). This initial study suggests no benefits to law enforcement administration of higher-dose naloxone were identified; more research is needed to guide public health agencies in considering whether 8-mg intranasal naloxone confers additional benefits for community organizations.

## Introduction

An 8-mg intranasal naloxone formulation, a higher-concentration product than had previously been available, was approved by the Food and Drug Administration (FDA) in 2021 for emergency treatment of known or suspected opioid overdose (*1*); however, no real-world data on use of the 8-mg product are available. The approval of the higher-concentration formulation was based on the 505(b)(2) approval pathway under the Federal Food, Drug, and Cosmetic Act, relying on data from the original FDA approval of naloxone (*1*) and supported by reports from both the FDA Advisory Committee (*2*) and the National Institutes of Health (*3*), which both suggested that higher-dose initial opioid reversal agents were needed to effectively respond to overdoses from synthetic opioids, including fentanyl. For example, one retrospective study of community members noted that the majority administered ≥2doses in responding to suspected overdoses (*4*)*. *However, no real-world quantitative data suggest that 4-mg intranasal naloxone is ineffective at reversing such overdoses.*

In 2014, New York began a law enforcement naloxone initiative, which includes developing and delivering training, and supplying naloxone to law enforcement, providing implementation guidance, and having a system for collecting data on naloxone administrations^†^ (*5*). The New York State Police (NYSP), a statewide law enforcement organization, reports the highest number of annual law enforcement naloxone administrations among New York law enforcement agencies, with approximately 360 reports per year (New York State Department of Health [NYSDOH], unpublished data, 2022). In New York, 4-mg intranasal naloxone is currently the product most commonly used by community responders, including law enforcement. For each person to whom naloxone is administered, law enforcement agencies submit a naloxone administration report to NYSDOH; reports include the following information: 1) date and time of administration, 2) age and perceived gender of the aided person, 3) county and zip code where the overdose occurred, 4) naloxone formulation used, 5) number of naloxone doses administered, 6) response to naloxone, 7) postnaloxone signs and symptoms, 8) emergency medical services disposition, and 9) survival.

Harm reduction advocates and medical professionals have noted potential harms of higher-dose naloxone, including severe withdrawal signs and symptoms, which can result in refusal of medical care, rapid reuse of opioids, reluctance to use naloxone if witnessing an overdose, and respiratory complications, including pulmonary edema and consequences of aspiration of vomitus (*6**,**7*). To evaluate this potential risk, in 2022, NYSDOH partnered with NYSP to field test 8-mg intranasal naloxone use by some NYSP troops. The aims of the study were to conduct real-world comparisons of survival, the average number of doses administered, presence of postnaloxone signs and symptoms, and hospital transport refusal among persons receiving the 8-mg or the 4-mg intranasal naloxone products.

## Methods

### Field Test: 8-mg versus 4-mg Intranasal Naloxone

In March 2022, NYSDOH partnered with NYSP to field test 8-mg intranasal naloxone by three of their 11 troops for use at the scene of a suspected opioid overdose. The three troops, located in eastern New York, received only 8-mg naloxone during this period. The other eight state police troops continued to receive the 4-mg intranasal product. All NYSP sworn members (state troopers) undergo standardized annual training on response to possible overdose events including patient assessment, naloxone use, and provision of rescue breathing. In addition, troopers receive biennial training in cardiopulmonary resuscitation, including chest compressions and automated external defibrillator usage. In 2022, the annual training included explanation of the field test and the change made to the reporting form to include dosage of intranasal naloxone administered. This study was reviewed and approved by the NYSDOH Institutional Review Board as non-research.^§^

The field test included a review of naloxone administration reports at regular team meetings, including by two physicians. When indicated, review of body-worn camera footage was conducted by study authors in collaboration with NYSP. Exclusion criteria included 1) absence of opioid toxidrome (i.e., respiratory depression or decreased consciousness), 2) more than one naloxone formulation (i.e., both 4-mg and 8-mg products) used by law enforcement responders, and 3) likely death before naloxone administration. Likely death before naloxone administration was ascertained by review of body-worn camera footage, responder reports, and defibrillator demonstration of asystole with no bystander cardiopulmonary resuscitation. 

### Data Analysis

Average number of naloxone doses administered per patient by formulation were compared using a t-test. Rates of survival and postnaloxone signs, symptoms, and behaviors (opioid withdrawal signs and symptoms including vomiting [reported as “dope sick” or “vomiting” by responders], lethargy, disorientation, perceived anger or combativeness, and hospital transport refusal) were compared using bivariate log-binomial regression for relative risk with associated p-values. Vomiting was also examined as an isolated postnaloxone sign separate from the grouped opioid withdrawal signs and symptoms variable. Persons who received the 4-mg intranasal naloxone product served as the referent group for all comparisons. P-values <0.05 were considered statistically significant. Analyses were conducted using SAS software (version 9.4; SAS Institute). 

## Results

### Naloxone Administration Reports

During March 26, 2022–August 16, 2023, NYSP troopers submitted 436 naloxone administration reports. After review, 354 (81.2%) forms met inclusion criteria, including 101 (29%) 8-mg and 253 (71%) 4-mg intranasal naloxone forms (Table). Overall, 99.0% of persons who received 8 mg and 99.2% of those who received 4-mg intranasal naloxone survived (relative risk [RR] = 0.81; p = 0.86). Recipients of 8-mg intranasal naloxone received an average of 1.58 doses (95% CI = 1.45–1.72), corresponding to a mean of 12.6 mg of naloxone. Recipients of 4-mg intranasal naloxone received an average of 1.67 doses (95% CI = 1.59–1.75), corresponding to a mean of 6.7 mg of naloxone. The mean number of doses administered per patient did not differ significantly by formulation (p = 0.27). Postnaloxone anger or combativeness as perceived by the responding law enforcement officer was reported in 11 of 101 (10.9%) 8-mg recipients and 20 of 253 (7.9%) 4-mg recipients and did not differ by formulation (RR = 1.42; p = 0.37). Most aided persons who were not deceased were transported to the hospital (75.6%; NYSDOH, unpublished data, 2022–2023), and hospital transport refusal did not differ significantly by formulation (RR = 0.65; p = 0.14).

**TABLE:** Reported outcomes and postnaloxone signs and symptoms among persons who received naloxone for suspected opioid overdose, by intranasal naloxone formulation as reported by New York State Police personnel (N = 354) — New York, March 2022–August 2023

Characteristic	Naloxone doses administered, no. (%)		RR (95% CI)	p-value for RR
	8 mg (n = 101)	4 mg* (n = 253)		
**Reported outcome**				
Survived	100 (99.0)	248 (99.2)	0.81 (0.07–8.99)	0.86
Perceived anger or combativeness	11 (10.9)	20 (7.9)	1.42 (0.66–3.09)	0.37
Refused transport to hospital	19 (19.0)	66 (26.6)	0.65 (0.36–1.15)	0.14
**Postnaloxone sign or symptom**				
Opioid withdrawal sign or symptom, including vomiting^†^	38 (37.6)	49 (19.4)	2.51 (1.51–4.18)	<0.001
Vomiting only	21 (20.8)	35 (13.8)	1.64 (0.90–2.98)	0.11
Disorientation	67 (66.3)	148 (58.5)	1.40 (0.86–2.27)	0.17
Lethargy	53 (52.5)	110 (43.5)	1.44 (0.90–2.28)	0.13

**Abbreviation:** RR = relative risk.

* Referent group.

^†^ New York training materials for law enforcement naloxone administration include nausea, vomiting, and withdrawal (sick feeling) as the key components of opioid withdrawal signs and symptoms for which to monitor after naloxone administration.

### Postnaloxone Signs and Symptoms

The most common postnaloxone signs and symptoms experienced among both groups were disorientation (8-mg recipients: 66.3%; 4-mg recipients: 58.5%) and lethargy (8-mg recipients: 52.5%; 4-mg recipients: 43.5%). RR for postnaloxone disorientation and lethargy did not differ significantly by formulation (p = 0.17 and 0.13, respectively).

Opioid withdrawal signs and symptoms including vomiting were significantly more prevalent among 8-mg naloxone recipients (37.6%) than among 4-mg recipients (19.4%), (RR = 2.51; p<0.001). Vomiting, one sign of withdrawal, was observed in 20.8% and 13.8% of 8-mg and 4-mg intranasal naloxone recipients, respectively; this was not significantly different by formulation (RR = 1.64; 95% CI = 0.90–2.98) (p = 0.11).

## Discussion

Despite the increased naloxone concentration in the 8-mg intranasal product, no significant differences were found in the survival of aided persons, or the number of doses administered by law enforcement by formulation, suggesting that, in this field test, the increased dosage did not provide added benefit, even in light of the increased prevalence of synthetic opioids, including fentanyl, in the drug supply.

Other studies have also found that number of naloxone doses administered in response to overdose has not changed over time, even with 4-mg and other lower-potency formulations (*8**,**9*). In this study, persons who received the 8-mg product were more than twice as likely to experience postnaloxone opioid withdrawal signs and symptoms including vomiting, compared with those who received the 4-mg intranasal naloxone product. When vomiting was analyzed as an isolated sign, no significant differences between formulations were found. However, the high prevalence of vomiting as an isolated sign in both groups is concerning because of the risk of aspiration in sedated persons.

### Limitations

The findings in this report are subject to at least four limitations. First, responding law enforcement personnel are not medical providers, and inconsistencies in their classification of postnaloxone symptoms or behaviors might have occurred. However, NYSP personnel have been reporting using a similar form for several years and are experienced in assessing symptoms and behaviors. Second, the number of 8-mg intranasal naloxone administration reports included was limited because only three of 11 NYSP troops received this formulation. With an increased sample size, additional differences in outcomes between groups might have been observed. Third, no information could be compared about differences between groups on the type or dose of substance used before suspected overdose, vital signs, or demographics. Finally, because the data were gathered from New York State only, the opioid potency might not reflect that in other areas.

### Implications for Public Health Practice

As reported in a 2022 review article (*7*), this study found no evidence supporting a benefit associated with administration of stronger opioid antagonists. In addition, the findings in this report align with data reported in a recent systematic review (*10*), which found that higher doses of naloxone administered in the emergency department were associated with a higher frequency of adverse events. This study is the first to provide real-world data comparing postnaloxone signs and symptoms and survival among persons administered 8-mg and 4-mg intranasal naloxone by community responders in response to a probable opioid overdose. This study suggests that there are no benefits to law enforcement administration of higher-dose naloxone. Additional data are needed to guide public health agencies in considering whether the 8-mg intranasal naloxone product provides benefits compared with the usual 4-mg intranasal naloxone product among community organizations, including law enforcement, given the lack of difference in survival rates or number of naloxone doses administered and the increased prevalence of opioid withdrawal signs and symptoms, including vomiting, in 8-mg recipients, when compared with recipients of 4-mg intranasal naloxone.

## Footnotes

* At the time of this writing, FDA has not approved intranasal naloxone doses >4 mg/0.1 mL for over-the-counter sales and has approved a lower dose (3 mg/0.1 mL) for such sales. https://www.fda.gov/news-events/press-announcements/fda-approves-second-over-counter-naloxone-nasal-spray-product^†^ New York training materials for law enforcement naloxone administration include nausea, vomiting, and withdrawal (sick feeling) as the key components of opioid withdrawal signs and symptoms for which to monitor after naloxone administration.

§ 45 C.F.R. part 46.101(c); 21 C.F.R. part 56.
